# Strengthening Cybersecurity for Patient Data Protection in Europe

**DOI:** 10.2196/48824

**Published:** 2023-08-24

**Authors:** Robin van Kessel, Madeleine Haig, Elias Mossialos

**Affiliations:** 1 LSE Health Department of Health Policy London School of Economics and Political Science London United Kingdom; 2 Institute of Global Health Innovation Imperial College London London United Kingdom

**Keywords:** cybersecurity, Europe, European Health Data Space, digital health, mHealth, medical informatics, data privacy, patient safety, privacy, health service

## Abstract

The health care sector experiences 76% of cybersecurity breaches due to basic web application attacks, miscellaneous errors, and system intrusions, resulting in compromised health data or disrupted health services. The European Commission proposed the European Health Data Space (EHDS) in 2022 to enhance care delivery and improve patients’ lives by offering all European Union (EU) citizens control over their personal health data in a private and secure environment. The EU has taken an important step in homogenizing the health data environment of the European health ecosystem, although more attention needs to be paid to keeping the health data of EU citizens safe and secure within the EHDS. The pooling of health data across countries can have tremendous benefits, but it may also become a target for cybercriminals or state-sponsored hackers. State-of-the-art security measures are essential, and the current EHDS proposal lacks sufficient measures to warrant a cybersecure and resilient environment.

Globally, 1463 cyberattacks were reported per week in 2022, with an average cost per breach of approximately US $10 million [1]. The European Union Agency for Cybersecurity (ENISA) reported that the health care sector in the European Union (EU) experienced 76% of cybersecurity breaches due to basic web application attacks, miscellaneous errors, and system intrusions in 2021. Internal threat actors also remained prominent, accounting for 39% of cybersecurity breaches [2]. The health care sector also faced numerous high-impact cybersecurity incidents that compromised sensitive data or disrupted health services, currently amounting to a median cost of €300,000 (US $325,000) per major security incident [3]. Additionally, the health care sector is one of the less mature sectors in the field of cybersecurity [4]. Disruptive attacks and lack of network segmentation allow foreign bodies to access the entire network instead of subsections, as well as exfiltrate sensitive information about the digital environment, which had a significant impact on the health sector. The societal migration to the digital world because of the COVID-19 pandemic worsened this situation, as fear and uncertainty among the public rose, resulting in a higher susceptibility to being exposed to harmful digital content and cybersecurity threats [5-8]. In fact, a 5-fold increase in cybercrimes was observed by the World Health Organization during the first 2 months of the pandemic [9]. This was further compounded by the distribution of counterfeit COVID-19 products on the dark web [10].

The European Commission proposed the European Health Data Space (EHDS) in 2022 to enhance care delivery and improve patients’ lives by offering all EU citizens control over their personal health data in a private and secure environment. The goal was to eliminate information barriers and establish a single market for digital health services [11]. More specifically, the EHDS enabled EU citizens to provide health professionals throughout the EU with access to their personal health data via a digital interface. This system would streamline the use of health data for purposes like research, innovation, policy making, and regulatory tasks, all the while upholding complete adherence to the EU’s data protection standards [11]. Rooted in the fundamental principles of civic participation and empowerment that define the EU, the EHDS also addresses obstacles hindering the broad acceptance of digital health methods in conventional health care [12,13]. Although concerns have been raised about the current iteration of the EHDS proposal, including its potential to exacerbate health inequalities instead of remedying them [14] and potential changes to data-sharing practices [15], the concept of cybersecurity has received limited attention.

The EHDS proposal only briefly mentions cybersecurity as a field that should be coordinated and collaborated with throughout the proposal (articles 10 [2], 39 [1], and 64 [5]) [5]. However, a global shortage of cybersecurity professionals in all domains was reported in a recent review on improving cybersecurity education [16]. One example of this is the recent release of the Cybersecurity Skills Academy by the European Commission, which was created to help close the cybersecurity talent gap and boost the EU’s competitiveness, growth, and resilience in cybersecurity [17]. Still, relying on a single supply source for cybersecurity professionals may be challenging due to significant labour market demand. One possible solution is to explore the feasibility of incorporating cybersecurity modules into medical, public health, and digital health curricula and providing retraining and upskilling opportunities for practicing professionals [16]. However, to achieve this, cybersecurity must be recognized and added as a core competency of digital health professionals, similar to what NHS Health Education England has implemented [18,19].

The EHDS proposal refers to the updated Network and Information Security Directive as a common cybersecurity framework [20], which requires EU member states to adopt various measures to improve their national cybersecurity environments. However, as a Directive, it leaves the responsibility to the member states to determine the means by which the objectives outlined in the Directive are achieved. This could pose a significant cybersecurity threat to the EU due to the divergent national cybersecurity strategies and resources allocated to achieve them [21]. EU member states with limited digital and cybersecurity capabilities, such as Southern or Eastern member states or small states, may be particularly vulnerable to coordinated attacks aimed at denying service and gaining unauthorized access [4,22]. To begin rectifying these disparities in digital infrastructure within the EU, one potential approach could involve the European Commission leveraging its extensive track record of infrastructure investments. This could entail establishing a dedicated investment portfolio aimed at extending digital infrastructure into underserved nations and communities.

A uniform cybersecurity system across the EU, and in particular, in the context of the EHDS, could provide a more comprehensive security net and enable the introduction of an EU-wide cybersecurity training curriculum. This system can include segmentation, multifactor authentication, and the use of virtual local area networks and cloud computing as well as training employees, monitoring behaviour, reducing human error, and enhancing stakeholder alignment [23,24]. Regarding segmentation, it is important to highlight that even though the system might be segmented on a national, regional, or local scale, all these segments would still operate within a unified federated network. In essence, despite being divided into numerous segmented networks, the system retains the ability to function as a cohesive, comprehensive network. In cases of cybersecurity threats, it is also feasible to isolate certain parts of the system to prevent the threat from infiltrating the federated network. Notably, recent technological advancements have demonstrated promising progress in the realm of cybersecurity. Technologies like blockchain [25-27] and a community solid server, which furnishes individuals with their personal data storage spaces [28], have been effective in addressing concerns related to patient privacy breaches. Furthermore, technologies such as Fast Healthcare Interoperability Resources (which dictate rules for exchanging electronic health care data) [29-31], the Observational Medical Outcomes Partnership Common Data Models (a standardized data format to facilitate consistent analysis of observational data) [32], and cross-enterprise document sharing (enabling cataloguing and sharing of patient records across health care institutions) are also capable of addressing cybersecurity issues tied to electronic health records [33]. Nevertheless, implementing effective cybersecurity measures may come at an additional cost; however, these costs are relatively insignificant compared to the direct costs of cybersecurity threats (mentioned above) and the potential direct and indirect repercussions of exposing the health data of EU citizens to substandard cybersecurity practices [2].

ENISA was created in 2019 to develop a high uniform level of cybersecurity within the EU and standardize and improve cybersecurity across its member states. However, its mandate is currently limited to providing technical and human resources to support EU member states, conducting reviews of cybersecurity policies and threats in the EU, and facilitating the exchange of best practices among the member states [22]. Although ENISA was established to enhance EU cybersecurity, the scope of its mandate prevents it from taking a leading role in the development of cybersecurity policies and resources. To achieve a homogeneous cybersecurity environment, ENISA's mandate needs to be expanded to proactively build and coordinate cybersecurity policies within EU member states and create a set of common cybersecurity standards. This would require the European Commission and EU member states to acknowledge the importance of addressing cybersecurity at the EU level [34]. By doing so, the EU can reduce potential negative consequences of divergent cybersecurity policies and build a stronger cybersecurity workforce to ensure the security of sensitive data within the EHDS and the health care sector. Additionally, this approach would enable ENISA to use its expertise and data related to cybersecurity threats. This could involve creating precise benchmarks for evaluating the affordability and sustainability of the EU cybersecurity system and assessing its cost-effectiveness—an area that currently lacks established criteria. Moreover, this strategy would empower ENISA to draw upon its industry-specific insights, enabling it to propose exemplary practices that harmonize with the digital capacities and preparedness of distinct industries.

The EU has taken a significant step toward homogenizing the health data environment in the European health ecosystem, but more attention is needed to ensure the safety and security of the health data of EU citizens within the EHDS. Although pooling health data across countries can bring tremendous public health benefits, it can also become a prime target for cybercriminals or state-sponsored hackers, posing a significant risk to EU citizens. Therefore, state-of-the-art security measures are essential, and the current iteration of the EHDS proposal does not contain sufficient measures to create a cybersecure and resilient environment.

## Abbreviations

EHDS: European Health Data Space
ENISA: European Union Agency for Cybersecurity
EU: European Union

## References

[1] Cost of a Data Breach Report 2023: fight back against data breaches. IBM.

[2] ENISA threat landscape 2022: July 2021 to July 2022. EU Publications.

[3] NIS investments: November 2022. EU PUblications.

[4] Tasheva I, Kunkel I (2022). In a hyperconnected world, is the EU cybersecurity framework connected?. European View.

[5] Williams CM, Chaturvedi R, Chakravarthy K (2020). Cybersecurity risks in a pandemic. J Med Internet Res.

[6] Alawida M, Omolara AE, Abiodun OI, Al-Rajab M (2022). A deeper look into cybersecurity issues in the wake of Covid-19: a survey. J King Saud Univ Comput Inf Sci.

[7] Holly L, Wong BLH, van Kessel R, Awah I, Agrawal A, Ndili N (2023). Optimising adolescent wellbeing in a digital age. BMJ.

[8] Roman-Urrestarazu A, Kinsey J, van Kessel R (2022). A bill of children's digital rights is required to improve and sustain children's futures globally. JAMA Pediatr.

[9] (2020). WHO reports fivefold increase in cyber attacks, urges vigilance. World Health Organization.

[10] Catalani V, Townshend HD, Prilutskaya M, Roman-Urrestarazu A, van Kessel R, Chilcott RP, Banayoti H, McSweeney T, Corazza O (2023). Profiling the vendors of COVID-19 related product on the Darknet: an observational study. Emerg Trends Drugs Addict Health.

[11] European Health Union: A European Health Data Space for people and science Internet. European Commission.

[12] Wong BLH, Maaß Laura, Vodden A, van Kessel R, Sorbello S, Buttigieg S, Odone A, European Public Health Association (EUPHA) Digital Health Section (2022). The dawn of digital public health in Europe: implications for public health policy and practice. Lancet Reg Health Eur.

[13] Marelli L, Stevens M, Sharon T, Van Hoyweghen I, Boeckhout M, Colussi I, Degelsegger-Márquez A, El-Sayed S, Hoeyer K, van Kessel R, Zając DK, Matei M, Roda S, Prainsack B, Schlünder I, Shabani M, Southerington T (2023). The European health data space: too big to succeed?. Health Policy.

[14] van Kessel R, Wong BLH, Forman R, Gabrani J, Mossialos E (2022). The European Health Data Space fails to bridge digital divides. BMJ.

[15] Shabani M (2022). Will the European Health Data Space change data sharing rules?. Science.

[16] AlDaajeh S, Saleous H, Alrabaee S, Barka E, Breitinger F, Raymond Choo K (2022). The role of national cybersecurity strategies on the improvement of cybersecurity education. Comput Secur.

[17] (2023). Cyber Skills Academy. Digital Skills & Jobs Platform.

[18] Artificial intelligence (AI) and digital healthcare technologies capability framework. NHS Health Education England: Digital Transformations.

[19] van Kessel R, Roman-Urrestarazu A, Anderson M, Kyriopoulos I, Field S, Monti G, Reed SD, Pavlova M, Wharton G, Mossialos E (2023). Mapping factors that affect the uptake of digital therapeutics within health systems: scoping review. J Med Internet Res.

[20] NIS2 Directive. European Commission.

[21] Markopoulou D, Papakonstantinou V, de Hert P (2019). The new EU cybersecurity framework: the NIS Directive, ENISA's role and the General Data Protection Regulation. CLSR.

[22] Martti L (2022). Cyber-attacks against critical infrastructure. Cyber Security.

[23] Stern AD, Gordon WJ, Landman AB, Kramer DB (2019). Cybersecurity features of digital medical devices: an analysis of FDA product summaries. BMJ Open.

[24] Kruse CS, Frederick B, Jacobson T, Monticone DK (2017). Cybersecurity in healthcare: a systematic review of modern threats and trends. THC.

[25] Roehrs A, da Costa CA, da Rosa Righi R (2017). OmniPHR: a distributed architecture model to integrate personal health records. J Biomed Inform.

[26] Madine MM, Salah K, Jayaraman R, Yaqoob I, Al-Hammadi Y, Ellahham S, Calyam P (2020). Fully decentralized multi-party consent management for secure sharing of patient health records. IEEE Access.

[27] Misbhauddin M, AlAbdulatheam A, Aloufi M, Al-Hajji H, AlGhuwainem A (2020). MedAccess: a scalable architecture for blockchain-based health record management.

[28] Celuchova Bosanska D, Huptych M, Lhotská L (2022). Decentralized EHRs in the semantic web for better health data management. Stud Health Technol Inform.

[29] Spratt S, Ravneberg D, Derstine B, Granger B (2022). Feasibility of electronic health record integration of a SMART application to facilitate patient-provider communication for medication management. Comput Inform Nurs.

[30] Lobach DF, Boxwala A, Kashyap N, Heaney-Huls K, Chiao AB, Rafter T, Lomotan EA, Harrison MI, Dymek C, Swiger J, Dullabh P (2022). Integrating a patient engagement app into an electronic health record-enabled workflow using interoperability standards. Appl Clin Inform.

[31] Bender D, Sartipi K (2013). HL7 FHIR: an agile and RESTful approach to healthcare information exchange.

[32] Standardized data: the OMOP common data model. OHDSI.

[33] Noumeir R, Renaud B (2010). IHE cross-enterprise document sharing for imaging: interoperability testing software. Source Code Biol Med.

[34] (2019). Regulation (EU) 2019/881 of the European Parliament and of the Council of 17 April 2019 on ENISA (the European Union Agency for Cybersecurity) and on information and communications technology cybersecurity certification and repealing Regulation (EU) No 526/2013 (Cybersecurity Act) (Text with EEA relevance) Internet. European Commission.

